# Physicochemical Properties of Berry Seeds Recovered from Pomace and Their Potential Applications in Food and Cosmetic Industries

**DOI:** 10.3390/ijms27010041

**Published:** 2025-12-19

**Authors:** Anna Kiełtyka-Dadasiewicz, Małgorzata Stryjecka, Aleksandra Głowacka, Živilė Tarasevičienė, Agata Jabłońska-Trypuć

**Affiliations:** 1 Department of Plant Production Technology and Commodity Sciences, University of Life Sciences, 20-950 Lublin, Poland; aleksandra.glowacka@up.lublin.pl; 2 Institute of Human Nutrition and Agriculture, The University College of Applied Sciences in Chełm, 22-100 Chełm, Poland; mstryjecka@panschelm.edu.pl; 3 Department of Plants Biology and Food Science, Faculty of Agronomy, Agriculture Academy Vytautas Magnus University, LT-44248 Kaunas, Lithuania; zivile.taraseviciene@vdu.lt; 4 Faculty of Civil Engineering and Environmental Sciences, Bialystok University of Technology, 15-351 Bialystok, Poland; a.jablonska@pb.edu.pl

**Keywords:** blackcurrant, strawberry, raspberry, nutrition, swelling index, color

## Abstract

The analysis of the physical and chemical properties of blackcurrant (*Ribes nigrum* L.), strawberry (*Fragaria ananassa* Duchesne ex Weston) and raspberry (*Rubus idaeus* L.) seeds recovered from pomace—food processing waste—was carried out. The weight of the one thousand seeds, their dry weight, swelling properties, and color in the CIE L*a*b* space, as well as the percentage of basic chemical components, i.e., protein, carbohydrate (including total dietary fiber, insoluble fiber, and soluble dietary fiber), fat, and ash were determined. Polyphenols content and antioxidant activity was determined. In addition, the amounts of individual phenolic compounds, fatty acids, and amino acids, as well as macro and micro-nutrients, were identified and analyzed. The potential usefulness of raspberry seeds as a rheology modifier of cosmetics and food products was estimated due to the high content of mucilage and swelling index similar to linseed and a favorable color with a high value of the b* parameter (22.1) corresponding to yellow color simultaneously with high luminescence (L* = 59.4). Oils obtained from all tested seeds are potentially useful in cosmetic preparations due to the high content of n-6 acids (50.4–71.5%), and oils from strawberry and raspberry seeds as a result of containing n-3 acids, respectively; 30.5–32.3% may be beneficial for dietary supplementation. In addition, the dietary values of the tested seeds are emphasized by the high content of dietary fiber (53.1–63.1%), antioxidant properties (the highest for blackcurrant) and the presence of phenolic compounds such as procyanidin derivatives, catechins (raspberry), quercetins and kaempferols (blackcurrant), and pelargonidin (strawberry).

## 1. Introduction

Processing of berry fruits in the food industry for juice, purée or fruit mousse production generates waste in the form of pomace, which besides peels and fibrous fruit fragments also contains seeds [1,2,3,4]. In the case of strawberries, due to their unique botanical structure, the so-called “seeds” are in fact true fruits (achenes), while the fleshy part is an accessory fruit derived from the enlarged receptacle [5,6]. Despite this botanical specificity, strawberries are treated as berry fruits in food processing because they share similar technological characteristics [7,8].

Seeds are considered the most valuable part of plants, as their function is to protect and provide nutrients required for the initial development of a new plant. However, in the production of delicate food products their presence is often undesirable due to their hard structure; therefore, seeds are removed during the manufacture of creamy purées and clear juices. Nonetheless, the oils contained in these seeds may constitute valuable raw materials with specific properties beneficial to the cosmetic industry [4,9,10]. Furthermore, the potential applications of pomace remaining after the extraction of seed oils from strawberries [11] and raspberries [2] have also been reported.

Poland is a key European producer and manufacturer of berry fruits [12,13]. Annual production reaches approximately 500 thousand tons, primarily consisting of strawberries (160–200 thousand t), raspberries (around 100 thousand t), and blackcurrants (120–140 thousand t). Global berry fruit production exceeds 12 million tonnes per year and is dominated by strawberries (ca. 10 million t), followed by currants (>0.7 million t), raspberries (~0.9 million t), and other berry fruits (~1 million t). In Europe, strawberries also predominate (ca. 1.7 million t), with currants (0.7 million t—nearly the entire global production) and raspberries (~0.6 million t) following [13].

More than half of polish raspberries, about 60% of strawberries, and over 70% of blackcurrants are processed, with juice production being particularly significant—especially for blackcurrants. Annually, approximately 25 thousand tons of strawberries (14% of yield), more than 20 thousand tons of raspberries (22%), and over 70 thousand tons of blackcurrants (54%) are directed to juice manufacturing [14]. It can therefore be estimated that over 120 thousand tons of soft fruits are processed solely into juice, with seeds also recoverable from purée production or further processing of frozen fruit. According to Martysiak-Żurowska and Drapała [15], seeds constitute around 1.5% of the mass of strawberries, 5.7% of raspberries, and ~5% of blackcurrants. This corresponds to an annual potential availability—exclusively from juice production—of approximately 0.37 thousand tons of strawberry seeds, 1.26 thousand tons of raspberry seeds, and 3.52 thousand tons of blackcurrant seeds in Poland. Seeds must be thoroughly separated from the wet pulp and peels, washed, and dried; for safe storage, moisture content should be below 12%.

Although often considered undesirable waste in the production of delicate fruit preparations, seeds may constitute a valuable source of raw materials for the cosmetic industry and various branches of the food sector—not only due to their nutritional value but also because of their physical properties [2,11]. Therefore, the aim of the present study was to evaluate the physicochemical characteristics and potential applications of seeds recovered as waste material after food processing of the three most common berry fruits in Poland [14]: strawberry, raspberry, and blackcurrant. To the best of our knowledge, this is the first work presenting such a comprehensive analysis of the bioactive components found in strawberry, raspberry, and blackcurrant seeds. The obtained results enabled us to identify potential directions for the utilization of these waste materials.

## 2. Results

The largest seeds were those of raspberry, with a mean thousand-seed weight of 1.78 g. Blackcurrant seeds were significantly lighter (1.17 g), while strawberry seeds were the smallest, with a thousand-seed weight of only 0.41 g (Table 1). Strawberry seeds exhibited the highest dry weight (DW) (94.3 g/100 g), whereas raspberry and blackcurrant seeds showed similar values of 92.5 and 93.2 g/100 g, respectively.

The swelling index was highest for raspberry seeds (5.52), significantly exceeding the values observed for strawberry (3.11) and blackcurrant (3.51). Comparative analyses performed for commercial swelling seeds showed values of 15.2 for chia seed and 6.0 for linseed. Notably, the swelling index of raspberry seeds did not differ statistically from that of linseed. These results were consistent with the mucilage content, which was also highest in raspberry seeds (2.49%), significantly surpassing that in strawberry (1.46%) and blackcurrant seeds (1.20%).

To the best of our knowledge, this is the first report providing color parameters of milled strawberry, raspberry, and blackcurrant seeds recovered as food-processing waste. In the CIE Lab* space, raspberry seed flour exhibited the highest luminosity (L* = 59.4), followed by strawberry (50.2), while blackcurrant seed flour was the darkest (L* = 33.4). A similar dependence was observed for the b* parameter (yellow–blue axis), with raspberry seeds showing the highest yellowness (b* = 22.1) and blackcurrant the lowest (b* = 7.1). It is worth noting that this was always a positive value, so they were characterized by a shade of yellow. The a* parameter (green–red axis) was highest in blackcurrant seeds (a* = 11.6), whereas strawberry and raspberry seeds showed statistically comparable values (8.7 and 8.8, respectively). Only in blackcurrant seeds did the red hue (a*) exceed the yellow hue (b*), giving the flour a more reddish tonality overall.

Carbohydrates constituted the largest group of macronutrients in the tested seeds (54.1–64.0%), most of which consisted of dietary fiber (53.1–63.1%). Soluble fiber predominated over insoluble fiber in all species (51.8–62.4% vs. 1.05–1.27%)—Table 2. Raspberry seeds contained the highest amounts of carbohydrates and fiber, whereas blackcurrant seeds contained the least. Proteins were another significant component in seeds. For blackcurrant (20.6%) and strawberry seeds (17.5%) it was the second group of compounds in terms of quantity. And for raspberries (proteins at 10.9%) it was the third, because their seeds contained more fat (14.2%).

Ash content ranged from 1.60% (strawberry) to 1.79% (blackcurrant), the level of which typically reflects the mineral content. Among the macro- and micronutrients assessed, blackcurrant seeds contained the highest amounts of potassium, phosphorus, magnesium, copper, and iron among the species tested. Strawberry seeds were the richest in calcium and manganese, while raspberry seeds generally contained the lowest amounts of macroelements but showed relatively higher levels of boron and zinc (Table 3).

Blackcurrant seeds exhibited the highest antioxidant potential, reflected in their total phenolic content (2679 mg GAE/100 g DW), carotenoid content (3.89 µg/g DW), and flavonoid content (94.2 mg CE/g DW). These values were significantly higher than those for raspberry and strawberry seeds (Table 4). Correspondingly, antioxidant activity assessed using DPPH and FRAP assays was also highest in blackcurrant seeds. Strawberry seeds consistently demonstrated the lowest antioxidant-related values.

The distribution of total anthocyanins differed from that of other polyphenols: raspberry seeds contained the highest anthocyanin levels (30.9 mg Pel-glc/g DW), followed by blackcurrant (23.7 mg Pel-glc/g DW) and strawberry (15.6 mg Pel-glc/g DW)—Table 5. Pelargonidin-3-glucoside was the predominant anthocyanin in all species, ranging from 8.21 mg/g DW (strawberry) to 23.7 mg/g DW (raspberry). Pelargonidin-3-malonylglucoside was the second-most abundant anthocyanin, with similar concentrations across species (4.28–4.59 mg/g DW). Other identified anthocyanins—cyanidin-3-O-glucoside, pelargonidin-3-rutinoside, cyanidin-3-malonylglucoside, and pelargonidin-3-acetylglucoside -were present at lower levels but were consistently highest in blackcurrant seeds.

**Figure 1:**
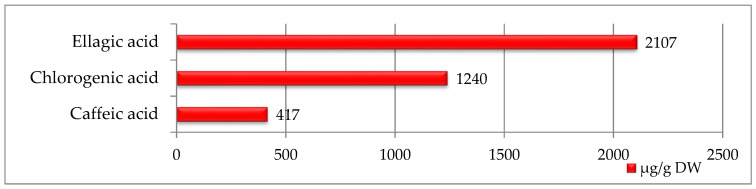
Phenolic compounds identified in strawberry seeds [μg/g DW].

In strawberry seed extracts, only three phenolic acids were identified, with ellagic acid being the most abundant (2107 µg/g DW) with the highest value among all seeds studied (Figure 1). Smaller amounts of chlorogenic acid (1240 µg/g DW) and caffeic acid (417 µg/g DW) were also detected.

**Figure 2:**
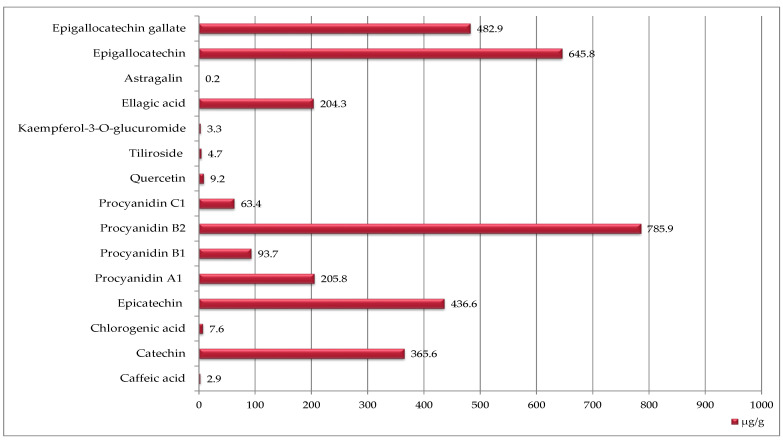
Phenolic compounds identified in raspberry seeds [μg/g DW].

Raspberry seeds contained a much broader spectrum of phenolic compounds—15 in total was determined (Figure 2). With procyanidin B2 being the most abundant (786 µg/g DW), followed by epigallocatechin, epigallocatechin gallate, epicatechin, catechin, procyanidin A1, and ellagic acid. However, the quantitative content of phenolic compounds in raspberry seeds was much lower than in strawberry seeds (Figure 1 and Figure 2).

**Figure 3:**
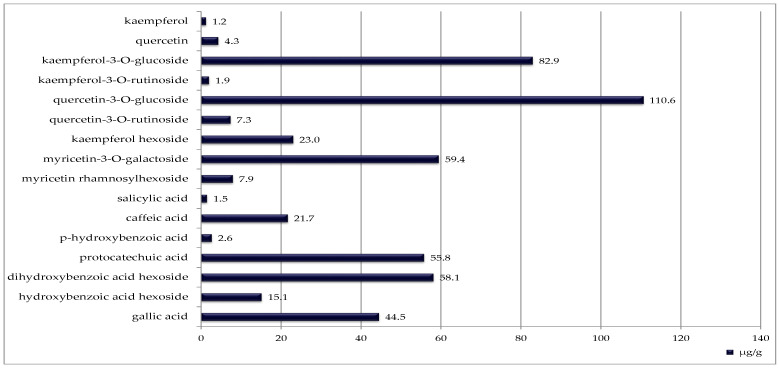
Phenolic compounds identified in blackcurrant seeds [μg/g DW].

Blackcurrant seeds contained the highest number of identified phenolic compounds (sixteen), although their concentrations were generally lower (Figure 3). The most abundant was quercetin 3-O-glucoside (110.6 µg/g DW).

Fatty acid profiles showed that linoleic acid (n-6) was the dominant fatty acid in all species, ranging from 46.0% in raspberry oil to 54.17% in strawberry oil (Table 6). The most pronounced difference between species was observed in linolenic acid (n-3) content, which was high in raspberry (32.2%) and strawberry seed oils (30.48%) but nearly absent in blackcurrant oil. Instead, blackcurrant oil contained substantial γ-linolenic acid (23.15%) from n-6 group. Consequently, total n-6 fatty acids were highest in blackcurrant seed oil (71.51%) and lower in strawberry (54.37%) and raspberry (50.4%) oils. In all species, polyunsaturated fatty acids accounted for the majority of total fatty acids (73.37–84.85%), whereas saturated fatty acids constituted only 3.42–8.55%.

Amino acid analysis demonstrated that glutamic acid was the most abundant essential amino acid, ranging from 1.17 mg/100 g (strawberry) to 1.48 mg/100 g (blackcurrant). High levels of aspartic acid, arginine, leucine, glycine, and other amino acids were also detected, with total amino acid content ranging from 6.38 mg/100 g (raspberry) to 7.06 mg/100 g (blackcurrant)—Table 7.

## 3. Discussion

The recovery of plant components from various branches of food processing aligns with the principles of the “zero waste” trend and is becoming increasingly common worldwide [1,2,3,4]. Saini et al. [1] describe numerous examples of how food-processing by-products can be valorized across different industries; however, the seeds of the berry fruits examined in our study are not mentioned, as their detailed composition has not previously been investigated in itself. The existing literature typically focuses on the characteristics of pomace remaining after the extraction of seed oil [16] or on fruit pomace (which includes seeds) left after juice production [17,18]. For this reason, the present study through conducting a comprehensive evaluation provides novel and valuable information on the functional properties and composition of strawberry, raspberry, and blackcurrant seeds recovered directly from food-processing pomace.

One of the most important functional properties of seeds considered as production additives—particularly in the cosmetic industry—are their rheological properties, which can be inferred from the swelling index and mucilage content. A high swelling index is also important in food applications, particularly in products designed to promote satiety, which are often used in weight-management diets. In our study, raspberry seeds exhibited the most favorable properties: their swelling index (5.52) was comparable to that of linseed (6.2) and exceeded the minimum requirement of the European Pharmacopoeia 9.0 [19] for milled linseed (not less than 4.5). Similar swelling capacities have been reported by Filipović et al. [20] for traditionally mucilaginous seeds such as marshmallow (5.0–6.0) and fenugreek (5.0–7.0), and slightly higher values have been reported for linseed (6.0–7.5). These findings suggest that raspberry seeds, often treated as waste, may represent an untapped resource for thickening or stabilizing formulations in both food and cosmetic products.

The color properties of milled raspberry seeds—characterized by high luminosity (L* = 59.4) and high yellowness (b* = 22.1)—were closest among the tested species to the optimal parameters of semolina used in pasta production, which requires a bright but intensely yellow color [21]. Thus, raspberry seed flour may be considered as a potential natural color-enhancing additive to flours derived from common wheat and other non-durum species, which typically exhibit lower b* values (13.2 for common wheat, 14.9 for spelt, and 16.3 for emmer) [22]. Even more favorable color parameters have been reported for milled rosehip seeds (L* = 68.9; b* = 26.2) [23]. In contrast, Ispiryan et al. [2] reported a darker color for whole raspberry seeds (L* = 44.6; b* = 13.3), which cannot be directly compared due to differences in measurement methodology. In their study, the color of raspberry seed after oil press—where seed structure is disrupted—showed a higher b* value (17.7 after cold pressing and >19 after CO_2_ extraction). There are no other literature references comparing the color of ground seeds. The Cie L*a* b* method is more common for determining the color of oils [24].

The chemical composition of the seeds revealed interspecific differences. Raspberry seeds had the highest carbohydrate (64%) and dietary fiber (62.4%) contents and relative to the other tested seeds high fat (14.2%) and small protein (10.9%) content. Similar oil content in raspberry seeds is reported by the other authors: Piasecka et al. [25] (14.25%); Dimić et al. [17] (about 14%); Pieszka et al. [26] (13.52%). Lower values are reported by Bada et al. [27] (10.55%) and Ispiryan et al. [2] (11.0%). Bederska-Łojewska et al. [28] observed a higher oil content (16.2%) in raspberry seeds, while the widest concentration range (12.1–20.%) is reported by Ispiryan et al. [9] depending on the raspberry variety. Other authors have previously described the dietary fiber values obtained from whole raspberry fruits [29] or from deoiled red raspberry pomace [30]. As far as we know, this is the first time we describe these characteristics in relation to the seeds themselves.

We found different proportions of basic nutrients for blackcurrant seeds than for raspberries. They had the highest carbohydrate content too (54.1%), including soluble fiber (51.8%), but contained more protein (20.6%) than fat (14.6%). We found similar relationships in the share of basic nutrients for strawberry seeds, which is consistent with the reports by Grzelak-Błaszczyk et al. [31], who reported that strawberry seeds can yield from 159 to 171 g/kg d.m. of proteins and more than 700 g/kg d.m. of total dietary fiber. Bada et al. 2013 [27] found a higher oil content in blackcurrant seeds than in our study, i.e., 26.15%, compared to 14.6%.

All seeds tested showed a high mineral content. In accordance with European nutrition legislation [32], a food may be considered a “significant source” of a mineral if 100 g of product provides at least 15% of the recommended daily intake. All tested seeds met this criterion for manganese (107–253%), iron (26–74%), magnesium (41–66%), phosphorus (35–88%), and zinc (25–27%). Additionally, blackcurrant seeds were a significant source of calcium (37%) and potassium (26%), while strawberry seeds were particularly rich in calcium (49%). Other authors also indicate blackcurrant seeds [33], raspberry seeds [1], and strawberry seeds [31] as good sources of micronutrients. However, Grzelak-Błaszczyk et al. [31] indicate significant variation in their content depending on the season. The high mineral content also confirms the importance of these seeds as dietary raw materials.

Polyphenols are the main compounds exhibiting antioxidant activity, so determining them can illustrate this activity. In our study, both total polyphenol content as gallic acid equivalent and both tests illustrating antioxidant activity (DPPH and FRAP) yielded the highest results for blackcurrant, and these values were significantly higher than those of both raspberry and strawberry seeds, for which the contents of total polyphenols, carotenoids, and flavonoids were the lowest. Ma et al. [34], similarly to our findings for seeds, indicate that blackcurrant fruit dominates in terms of GAE equivalent content over both strawberries and blackcurrant fruit, as well as the other berries tested. Helbig et al. [16] do not confirm the superiority of blackcurrant over strawberry, as they report similar GAE equivalent content, but in pomace after oil press at 60 °C, which may alter the composition of active ingredients and antioxidant capacity. Total anthocyanins were highest in raspberry seeds and lowest in strawberries. Similar or slightly lower (9.8–15.2 mg/100 g DW depending on the season) anthocyanin content in deffated strawberry seeds is reported by Grzelak-Błaszczyk et al. [31].

In our studies, we identified six anthocyanins in the seeds of all tested fruits: pelargonidin-3-glucoside (8.21–23.7 mg/g DW), pelargonidin-3-malonylglucoside (4.28–4.59 mg/g DW), cyanidin-3-O-glucoside (1.79–2.04 mg/g DW), cyanidin-3-malonylglucoside (0.68–0.77 mg/g DW), pelargonidin-3-rutinoside (0.28–0.37 mg/g DW), and pelargonidin-3-acetylglucoside (0.10–0.18 mg/g DW). There is no data in the available literature on the content of anthocyanins in seeds, but many authors confirm their presence in fruits, although in such a situation their amounts cannot be directly compared. Aaby et al. [35] confirm the presence of the same compounds in strawberry fruit, and that, similarly to our results, the dominant anthocyanin was pelargonidin-3-glucoside. However, quantitative comparisons cannot be made directly due to differences in biological material (seeds vs. whole fresh fruit) and moisture content. Furthermore, the literature data indicate the possibility of variation in anthocyanin content depending on strawberry genotype [35]. Staszowska-Karkut and Materska [36] confirm the presence of the phenolic compositions we identified in blackcurrant and raspberry leaves.

Determining the fatty acid composition of oil was not the primary goal of our analyses; as a technological innovation, we wanted to indicate other potential directions of seed processing and their intended use as an agri-waste source. The fatty acid composition has been studied and extensively described by many authors for all the species discussed: strawberry [3,26,37,38], blackcurrant [3,4,26,38], and raspberry [9,26,38,39]. In this study, we confirmed the high content of polyunsaturated acids, including linoleic acid and linolenic acid, and the low content of saturated acids, which is typical for oils from fruit plant seeds [10,23,40,41,42].

## 4. Materials and Methods

### 4.1. Plant Materials

**Figure 4:**
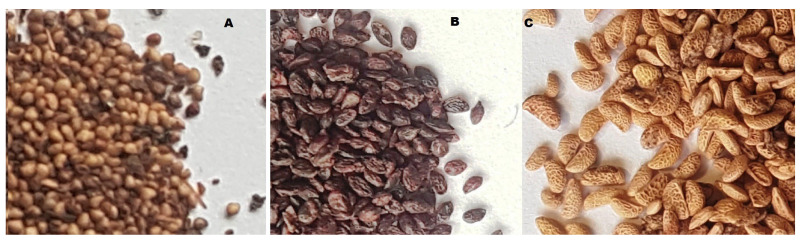
Photos of seeds used for the research (**A**). Strawberry, (**B**). Blackcurrant, (**C**). Raspberry.

The plant material consisted of seeds recovered in 2021 during food-processing operations of three berry fruit species: blackcurrant (*Ribes nigrum* L.), strawberry (*Fragaria × ananassa* Duchesne ex Weston), and raspberry (*Rubus idaeus* L.). The seeds were obtained from a fruit-processing facility producing juices, with the producer declaring Poland as the country of origin of the fruit (H.J.C. Polecki Trading, Śrem, Poland). The purchased seeds had been separated from pomace, rinsed, and dried by the producer prior to delivery (Figure 4).

### 4.2. Physicochemical Parameters

The mass of 1000 seeds (g) was determined using random samples of clean, air-dried seeds. From each species, 200 seeds were counted and weighed to the nearest 0.01 g, and the results were recalculated to 1000 seeds. Measurements were performed in four replications for each species.

The dry weight (DW) content of seeds was determined after milling by drying at 105 °C for at least 4 h to constant weight.

The swelling index was determined according to the European Pharmacopoeia 9.0 [19]. One gram of milled seeds was placed in a 25 mL ground-glass stoppered cylinder (height 125 ± 5 mm, 0.5 mL graduations). Subsequently, 25 mL of water was added, the cylinder was closed, and the mixture was shaken vigorously every 10 min for 1 h. The samples were then incubated for 3 h. Ninety minutes after the beginning of the test, trapped air and floating seed particles were released by rotating the cylinder around its vertical axis. The volume occupied by the swollen material—including adhering mucilage—was recorded. Three parallel tests were performed.

Mucilage content was determined according to a modified procedure described by Lai and Liang [43] and Odep et al. [44]. A 1 g portion of ground seeds was placed in an Erlenmeyer flask and extracted with 100 mL of distilled water. The mixture was heated in a water bath at 60 °C for 1 h, with manual stirring every 10 min to facilitate the extraction of polysaccharide fractions. After cooling, the extract was filtered sequentially through gauze and Whatman No. 1 filter paper. The filtrate was transferred into pre-dried, pre-weighed evaporating dishes and dried at 60 °C to constant weight (36 h).

Mucilage content was expressed as a percentage of dry weight (DW) according to the following equation:

$$Mucilage\;content\;\left(\%\;DW\right)\;=\;\frac{m\;dry\;residue}{m\;sample\;(DW)}\;\times \;100$$

where

m dry residue—mass of the dried mucilage after evaporation (g),

m sample (DW)—mass of the dry sample prior to extraction (g).

Color measurements were performed using a Neo9000 spectrophotometer (3Color^®^, Kraków, Poland) in the CIE Lab* system with Spectro Color QC software (V2.79.0.5.20201209). Measurements were taken in reflected light, SCE mode, with illuminant D65/10°.

### 4.3. Basic Biochemical Parameters

Seeds were dried to constant weight in a convection oven at 105 °C, ground in a laboratory mill, and homogenized. All analyses were performed on material expressed on a dry-weight basis (DW). Each measurement was carried out in triplicate, and the results are reported as mean ± SD.

The contents of protein, fat, ash, and carbohydrates in seeds were determined according to standard proximate methods. Crude protein was quantified using the Kjeldahl method [45] (ISO 5983; conversion factor N × 6.25). Crude fat was determined by Soxhlet extraction with n-hexane following AOAC 920.39 [46]. Ash content was measured by incineration in a muffle furnace at 550 °C according to AOAC 942.05 [47]. Total carbohydrates were calculated by difference: 100—(protein + fat + ash + moisture). All results were expressed on a dry-weight basis (DW). Analyses were conducted in triplicate and reported as mean ± SD.

### 4.4. Polyphenols and Antioxidant Activity

#### 4.4.1. Extraction Procedure

Extracts from powdered seeds were prepared to determine total phenolic content and antioxidant activity as below. The ground seeds were dried to a constant weight and homogenized prior to analysis. To prepare the extracts, 0.50 ± 0.01 g of the dried sample was transferred to 15 mL Falcon tubes and extracted with 10 mL of 80% methanol (*v*/*v*) acidified with 1% acetic acid. The samples were sonicated for 20 min at room temperature (22–25 °C) in an ultrasonic bath to improve the extraction efficiency of phenolic compounds. After sonication, the samples were centrifuged at 5000× *g* for 10 min, and the supernatant was collected in volumetric flasks and made up to 10 mL with extraction solvent. The extracts were filtered through 0.45 μm nylon membrane filters and stored at 4 °C until analysis. All extracts were prepared in triplicate.

#### 4.4.2. Total Phenolic Content (TPC)

Total phenolic content was determined using the Folin–Ciocalteu method as described by Singleton and Rossi [48], with minor modifications. An aliquot of 0.1 mL of extract was mixed with 1.0 mL of diluted Folin–Ciocalteu reagent (1:10, *v*/*v*). After 5 min of incubation, 1.5 mL of 10% (*m*/*v*) sodium carbonate solution was added to provide alkaline conditions for the reduction reaction. The mixture was vortexed and incubated in the dark for 60 min at room temperature. Absorbance was measured at 765 nm using a UV2600i-plus spectrophotometer (Shimadzu, Kyoto, Japan).

Calibration was performed using gallic acid standard (Sigma-Aldrich, St. Louis, MO, USA, 97.5–102.5% titration) solutions (0–200 mg·L^−1^). Results were expressed as mg gallic acid equivalents per g dry weight (mg GAE·g^−1^ DW). All measurements were performed in triplicate.

#### 4.4.3. DPPH Radical-Scavenging Activity

The antioxidant activity was evaluated using the DPPH radical-scavenging assay as described by Brand-Williams et al. [49], with minor modifications. A total of 3.943 mg of DPPH (weighed with an accuracy of ±0.01 mg) was added to a 100 mL volumetric flask containing approximately 100 mL of methanol (HPLC-grade). The compound was dissolved using gentle mixing (rotary mixer and/or brief ultrasonic bath) until the colored suspension was completely dissolved, and the solution was then made up to the mark with methanol. The DPPH solution was stored in an amber glass bottle or wrapped in aluminum foil at 4 °C and used within 24–48 h due to its light sensitivity and potential gradual degradation. The solution was prepared immediately before analysis when maximal stability was required. An aliquot of 0.1 mL of extract was mixed with 3.9 mL of the DPPH solution and incubated for 30 min in the dark at room temperature. Absorbance was measured at 517 nm using a UV2600i-plus spectrophotometer (Shimadzu, Japan). The percentage of DPPH inhibition was calculated, and antioxidant capacity was expressed as µmol Trolox·g^−1^ DW based on a calibration curve prepared from Trolox standards (0–500 µM). All measurements were performed in triplicate.

#### 4.4.4. FRAP Assay

The ferric reducing antioxidant power (FRAP) was determined according to Benzie and Strain [50], with minor modifications. The FRAP reagent was prepared freshly by mixing 300 mM acetate buffer (pH 3.6), 10 mM TPTZ in 40 mM HCl, and 20 mM FeCl_3_·6H_2_O (10:1:1, *v*/*v*/*v*). A total of 0.1 mL of extract was added to 3.0 mL of the FRAP reagent and incubated at 37 °C for 30 min in the dark. Absorbance was recorded at 593 nm (UV2600i-plus, Shimadzu, Japan). Calibration was performed using Trolox solutions (0–1000 µM), and results were expressed as µmol Trolox·g^−1^ DW. All measurements were conducted in triplicate.

#### 4.4.5. Total Carotenoid Content

Total carotenoid content was determined spectrophotometrically according to the method described by Lichtenthaler and Buschmann [51], with minor modifications. Samples were homogenized in 80% (*v*/*v*) acetone at a ratio of 1:20 (*m*/*v*) and extracted in the dark for 20 min at 4 °C. After centrifugation (5000× *g*, 10 min), the clear supernatant was transferred to a quartz cuvette, and absorbance was measured at 470 nm using 80% acetone as the blank.

#### 4.4.6. Total Flavonoid Content (Colorimetric AlCl_3_ Method)

Total flavonoid content was determined using the colorimetric aluminum chloride complexation method, according to the procedure described by Chang et al. [52], with minor modifications. To 1 mL of the extract, 0.3 mL of a 5% (*m*/*v*) NaNO_2_ solution was added. After 5 min, 0.3 mL of a 10% (*m*/*v*) AlCl_3_ solution was added, followed by 2 mL of 1 mol·L^−1^ NaOH after an additional 6 min. The mixture was then diluted with distilled water to a final volume of 10 mL, thoroughly mixed, and the absorbance was measured at 510 nm using a UV2600i-plus spectrophotometer, with distilled water serving as the blank.

The total flavonoid concentration was quantified using a calibration curve prepared from quercetin standards (Sigma-Aldrich, ≥95%) (range: 0–100 mg·L^−1^). The results were expressed as milligrams of quercetin equivalents per gram of dry weight (mg QE·g^−1^ DW):

$$Total\;flavonoids\;\left(mg\;QE\;g^{-1}\;DW\right)\;=\;\frac{mg\;of\;quercetin\;equivalents\;in\;the\;extract}{g\;of\;dry\;weight\;of\;the\;sample}$$

#### 4.4.7. Total Anthocyanin Content

The total anthocyanin content was determined using the pH differential method as described by Giusti and Wrolstad [53]. This method is based on the structural transformation of anthocyanins, which predominantly exist in the colored flavylium cation form at pH 1.0, whereas at pH 4.5 they are largely converted into the colorless hemiketal form. The difference in absorbance between these two protonation states is proportional to the anthocyanin concentration in the sample. 

The sample was mixed with potassium chloride buffer at pH 1.0 (0.025 mol·L^−1^) and sodium acetate buffer at pH 4.5 (0.4 mol·L^−1^) in a 1:9 ratio (*v*/*v*). Following a 15 min incubation at room temperature, absorbance was measured at 520 nm and 700 nm (to correct for sample haze). The absorbance difference was calculated using the following equation:


$$A\;=\;{\left(A_{520}\;-\;A_{700}\right)}_{pH\;1,0}\;-\;{\left(A_{520}\;-\;A_{700}\right)}_{pH\;4,5}$$

The total anthocyanin content was expressed as cyanidin-3-glucoside (C3G) equivalents according to the following formula:

$$\mathrm{Anthocyanin}\;(\mathrm{mg}/L)\;=\;\frac{A\times MW\times DF\times 1000}{\varepsilon \times l}$$

where

- MW = 449.2 g·mol^−1^ (molecular weight of cyanidin-3-glucoside),
- DF—dilution factor,
- ε = 26,900 L·mol^−1^·cm^−1^ (molar extinction coefficient for C3G),
- l = 1 cm (optical path length).

The final results were expressed as mg total anthocyanins per g of dry weight (mg·g^−1^ DW), taking into account the sample mass and all dilution steps.

#### 4.4.8. Identification and Quantification of Phenolic Compounds

Phenolic compounds presented in Figure 1, Figure 2 and Figure 3 were identified and quantified using high-performance liquid chromatography with diode-array detection (HPLC-DAD) following the procedure described by González-González et al. [54], with minor modifications adapted to the analysed matrix. Extraction was carried out according to the procedure described in Section 4.3, using 80% methanol (*v/v*) acidified with 1% acetic acid. Analyses were performed using a Prominence-1 LC-2030C 3D HPLC system (Shimadzu, Tokyo, Japan). The separation was carried out on a reversed-phase C18 column (250 × 4.6 mm, 5 µm). The mobile phase consisted of solvent A: water with 0.1% formic acid, and solvent B: acetonitrile.

The gradient elution was as follows: 0–5 min, 5% B; 5–40 min, linear increase to 40% B; 40–45 min, return to 5% B. The flow rate was 1.0 mL·min^−1^, column temperature 30 °C, and injection volume 20 µL. UV-Vis spectra were recorded in the range of 200–600 nm, and chromatograms used for quantification were monitored primarily at 280 nm (phenolic acids and flavan-3-ols), 320 nm (hydroxycinnamic acids), and 360 nm (flavonols).

Individual phenolic acids, flavan-3-ols, flavonols, and other phenolic compounds were identified based on comparison of retention times with analytical standards. UV–Vis spectral matching using diode-array detection.

Co-chromatography with selected standards when needed. Authentic reference standards (Sigma-Aldrich, ≥95% purity) were used for ellagic acid, chlorogenic acid, caffeic acid, catechin, epicatechin, epigallocatechin, epigallocatechin gallate, procyanidin A1, procyanidin B2, quercetin-3-O-glucoside, kaempferol derivatives, and other detected compounds.

Quantification was performed using external calibration curves prepared from the corresponding standards (concentration range 0.5–50 mg·L^−1^; r^2^ ≥ 0.999). For compounds without commercially available standards, quantification was performed using calibration curves of structurally related compounds with similar chromophores (e.g., catechin for other flavan-3-ols).

Concentrations were calculated based on peak areas and expressed as µg·g^−1^ dry weight (DW) of sample. All measurements were performed in triplicate, and results are reported as mean ± SD.

### 4.5. Determination of the Qualitative and Quantitative Composition of Fatty Acids

Lipids were extracted using the chloroform–methanol method according to ISO standards [55]. To 1.0 g of homogenized sample, 20 mL of a chloroform/methanol mixture (2:1, *v*/*v*) was added, followed by vigorous mixing for 10 min. Subsequently, 4 mL of 0.9% NaCl solution was added to facilitate phase separation. After separation, the chloroform layer was collected, and the solvent was evaporated to dryness under reduced pressure. The dry lipid residue was then dissolved in a defined volume of chloroform for further analyses [55].

Fatty acid methyl esters (FAME) were prepared using a direct transesterification procedure [56]. For this purpose, 50 mg of lipid extract was transferred into a reaction vial containing 2 mL of methanol with 2% H_2_SO_4_ (*v*/*v*) as the catalyst and 1 mL of hexane (for FAME extraction). The mixture was heated at 80 °C for 1 h, then cooled and diluted with water. The organic layer containing FAME was isolated, dried over anhydrous Na_2_SO_4_, and evaporated to dryness under nitrogen.

Analyses were performed using gas chromatography with flame ionization detection (GC-FID) (GC-2010 Pro, Shimadzu, Japan). The chromatographic conditions were as follows: a capillary column suitable for FAME analysis (e.g., SP-2560 or equivalent, 100 m × 0.25 mm, 0.20 µm film thickness); carrier gas—helium at a flow rate of 1.0 mL·min^−1^; injection volume—1 µL; split mode (1:50). Temperature settings: injector at 250 °C, detector (FID) at 260 °C. Oven program: 100 °C (2 min), increased at 10 °C·min^−1^ to 180 °C, then at 3 °C·min^−1^ to 230 °C, held at 230 °C for 10–15 min. Fatty acids were identified by comparing retention times with commercial FAME standard mixtures (covering C4–C24). Identification was confirmed by matching analyte retention times with those of reference standards.

Quantification was performed using the internal standard method. Prior to transmethylation, a known concentration of an internal standard (e.g., heptadecanoic acid methyl ester, C17:0) was added to each sample. The content of individual fatty acids was calculated based on the ratio of the analyte peak area to the internal standard peak area.

### 4.6. Amino Acid Profile Determination

The qualitative and quantitative composition of amino acids was determined using high-performance liquid chromatography (HPLC) (Prominence-1 LC-2030C 3D HPLC system, Shimadzu, Tokyo, Japan) following acid hydrolysis, according to the AOAC method 982.30 procedure [57]. Samples were hydrolyzed in 6 mol·L^−1^ HCl under a nitrogen atmosphere at 110 °C for 24 h. After hydrolysis, the solutions were evaporated under reduced pressure and reconstituted in phosphate buffer.

Prior to chromatographic analysis, amino acids were derivatized: primary amino acids reacted with o-phthalaldehyde (OPA), while secondary amino acids were derivatized using FMOC reagent. Chromatographic separations were performed using an HPLC system equipped with a C18 reversed-phase column and a fluorescence detector. A gradient elution program was applied using phosphate buffer as mobile phase A and an acetonitrile–methanol mixture as mobile phase B. Amino acids were identified based on comparisons of retention times with those of external standards.

Amino acid separation was conducted on a C18 reversed-phase column (150 × 4.6 mm, 5 µm). The column temperature was maintained at 40 °C, and the injection volume was 10 µL. The mobile phase flow rate was set at 1.0 mL·min^−1^. Gradient elution was performed with two mobile phases:

- Phase A: 40 mmol·L^−1^ phosphate buffer (pH 7.8)
- Phase B: acetonitrile/methanol/water (45:45:10, *v*/*v*/*v*)

The total analysis time was 45 min.

Quantification was carried out by external calibration using standard amino acid solutions of known concentrations. Sulfur-containing amino acids (cysteine and methionine) were determined following prehydrolytic oxidation (formic acid/hydrogen peroxide) to their stable oxidation products—cysteic acid and methionine sulfone. Results were expressed as mg of amino acid per g of dry matter. All analyses were performed in triplicate.

### 4.7. Mineral Components Determination

Seeds were digested in analytically pure nitric acid (HNO_3_). The contents of calcium (Ca), potassium (K), phosphorus (P), magnesium (Mg), copper (Cu), manganese (Mn), iron (Fe), zinc (Zn), and boron (B) were determined by atomic absorption spectrometry (AAS) according to EN-ISO 6869:2000 [58], using a SOLAR 939 (Unicam) spectrometer (Camberley, Surrey, UK). Results were expressed as mg/kg dry weight (DW).

## 5. Conclusions

The results of our study provide a basis for considering the examined seeds as food additives that serve as sources of dietary fiber and protein, particularly blackcurrant and strawberry seeds. Raspberry seeds, due to their highest content of dietary fiber, mucilages, and their high swelling capacity, may be considered as an additive to dietary products supporting weight-loss processes. They may also be useful in food products for which a yellow coloration is essential, such as pasta. All analyzed seed species proved to be good sources of minerals—especially manganese, iron, magnesium, phosphorus, and zinc—which confirms their high nutritional value. In addition, the dietary benefits of the studied seeds are highlighted by their high dietary fiber content (53.1–63.1%), antioxidant properties (highest in blackcurrant), and the presence of phenolic compounds such as procyanidin derivatives, catechins (raspberry), quercetin and kaempferol (blackcurrant), and pelargonidin (strawberry).

Due to their high mucilage content and a swelling capacity comparable to that of flaxseed, raspberry seeds may be considered for their potential role as rheology modifiers in cosmetic, food, and other products in which consistency is important. It is also worth emphasizing their favorable color, characterized by a high b* value (22.1), corresponding to yellow coloration, along with high lightness (L* = 59.4).

Oils extracted from all three seed types may be useful ingredients in cosmetic preparations due to their high content of n-6 fatty acids (50.4–71.5%). For dietary supplementation, strawberry and raspberry seed oils are particularly promising because of their high levels of n-3 fatty acids (30.5–32.3%).

The findings also provide a solid foundation for the development of future research models involving the incorporation of berry seed ingredients into products such as pa-sta, cookies, emulsions, or cosmetic creams. Owing to their content of anthocyanins, phenols, phenolic acids, carotenoids, minerals and other bioactive compounds, these ingredients may also enrich human diets with functional components.

## Figures and Tables

**Table 1:** Physical parameters of tested berry fruit seeds.

Parameters:		Seeds	
	Strawberry	Raspberry	Blackcurrant
Mass of 1000 seeds [g]	0.41 c	1.78 a	1.17 b
Dry weight (DW) [g/100 g]	94.3 a	92.5 b	93.2 b
Swelling index ^#^	3.11 b	5.52 a	3.51 b
Mucilage [%]	1.46 b	2.49 a	1.20 b
Color parameters ^#^:			
L*	50.2 b	59.4 a	33.4 c
a*	8.7 b	11.6 a	8.8 b
b*	15.4 b	22.1 a	7.1 c

^#^ after milling; L*—parameter indicates luminance (the higher the value, the lighter the sample), a*—parameter indicates from green (negative value) to red color (positive value), b*—parameter indicates from blue (negative value) to yellow color (positive value). Values marked with the same letter in rows do not differ significantly at *p* > 0.05.

**Table 2:** Basic biochemical parameters of tested berry fruit seeds.

Parameters		Seeds	
	Strawberry	Raspberry	Blackcurrant
Protein [%]	17.5 b	10.9 c	20.6 a
Carbohydrate [%]	63.6 a	64.0 a	54.1 b
Total dietary fiber [%]	60.9 b	63.1 a	53.1 c
Insoluble fiber [%]	1.05 a	1.16 a	1.27 a
Soluble dietary fiber [%]	59.9 b	62.4 a	51.8 c
Fat [%]	10.2 b	14.2 a	14.6 a
Ash [%]	1.60 c	1.70 b	1.79 a

Values marked with the same letter in rows do not differ significantly at *p* > 0.05.

**Table 3:** Macro- and micro-elements in berry fruit seeds.

Seeds	Mineral Content [mg/kg]								
	Ca	K	P	Mg	Cu	Mn	Fe	Zn	B
Strawberry	3910 a	2050 b	4780 b	1800 b	8.1 b	50.5 a	36.5 c	25.0 b	7.09 a
Raspberry	1130 c	1600 c	2450 c	1550 c	7.0 c	36.8 b	65.0 b	26.8 a	7.34 a
Blackcurrant	2960 b	5110 a	6200 a	2490 a	10.6 a	21.5 c	104 a	26.1 a	6.19 b

Values marked with the same letter in columns do not differ significantly at *p* > 0.05.

**Table 4:** Polyphenols and antioxidant activity of tested berry fruit seeds.

Parameters		Seeds	
	Strawberry	Raspberry	Blackcurrant
Total phenols [mgGAE/100 g DW]	1540 c	1771 b	2679 a
DPPH [mg TE/g DW]	1215 c	1418 b	1959 a
FRAP [mg TE/g DW]	2507 c	2916 b	3658 a
Carotenoids [µg/g DW]	2.20 c	2.79 b	3.89 a
Flavonoids [mg CE/g DW]	88.7 c	90.0 b	94.2 a

Values marked with the same letter in rows do not differ significantly at *p* < 0.05.

**Table 5:** Total and particular anthocyanins of tested berry fruit seeds.

Anthocyanins		Seeds	
	Strawberry	Raspberry	Blackcurrant
Total [mg Pel-glc/g DW]	15.6 c	30.9 a	23.7 b
Particular anthocyanins [mg/g DW]:			
Cyd-3-glu	1.95 b	1.79 c	2.04 a
Pg-3-glu	8.21 c	23.7 a	15.7 b
Pg-3-rut	0.34 a	0.28 b	0.37 a
Cyd-3-malglu	0.71 b	0.68 b	0.77 a
Pg-3-malglu	4.28 a	4.41 a	4.59 a
Pg-3-acetglu	0.10 b	0.10 b	0.18 a

Values marked with the same letter in rows do not differ significantly at *p* < 0.05.

**Table 6:** Fatty acid content (%) from berry fruit seed oils.

Fatty Acid		Seeds		
		Strawberry	Raspberry	Blackcurrant
Myristic acid	14:0	0.00	0.00	0.16
Palmitic acid	16:0	2.16	2.25	6.44
Palmitoleic acid	16:1 (n-7)	0.20	0.39	0.15
Hexadecadienoic acid	16:2 (n-6)	0.10	0.15	0.00
Stearic acid	18:0	0.76	0.85	1.53
Oleic acid	18:1 (n-9)	10.81	12.22	16.48
Vaccenic acid	18:1 (n-7)	0.52	0.71	0.66
Linoleic acid	18:2 (n-6)	54.17	46.01	48.36
γ-linolenic acid	18:3 (n-6)	0.10	4.24	23.15
Linolenic acid	18:3 (n-3)	30.48	32.20	0.01
Stearidonic acid	18:4 (n-3)	0.00	0.00	1.85
Arachidic acid	20:0	0.40	0.23	0.20
Eicozeic acid	20:1 (n-9)	0.20	0.60	1.00
Behenic acid	22:0	0.10	0.15	0.20
Ʃ SFA		3.42	3.48	8.55
Ʃ MUFA		11.73	13.92	18.08
Ʃ PUFA		84.85	82.6	73.37
Ʃ n-3		30.48	32.2	1.86
Ʃ n-6		54.37	50.4	71.51
Ʃ n-7		0.72	1.1	0.76
Ʃ n-9		11.01	12.82	17.32

Ʃ—sum; SFA—fatty acids without double bonds (..:0); MUFA—fatty acids with one double bond (..:1); PUFA—fatty acids with two or more double bonds (..:2) (…:3) (…:4).

**Table 7:** Amino acid content [mg/100 g] from berry fruit seeds.

Amino Acid Content [mg/100 g]		Seeds	
	Strawberry	Raspberry	Blackcurrant
glutamic acid	1.43	1.17	1.48
aspartic acid	0.87	0.75	0.78
arginine	0.61	0.52	0.56
leucine	0.47	0.48	0.54
glycine	0.41	0.40	0.49
alanine	0.36	0.33	0.32
valine	0.37	0.34	0.35
isoleucine	0.28	0.31	0.26
lysine	0.38	0.30	0.37
phenylalanine	0.24	0.28	0.30
proline	0.26	0.27	0.25
serine	0.28	0.26	0.32
threonine	0.25	0.24	0.29
histidine	0.18	0.18	0.20
cysteine	0.18	0.17	0.16
tyrosine	0.13	0.13	0.12
methionine	0.18	0.15	0.16
hydroxyproline	0.03	0.04	0.03
tryptophan	0.05	0.04	0.06
taurine	0.01	0.01	0.00
ornithine	0.01	0.01	0.00
sum:	6.97	6.38	7.06

## Data Availability

The original contributions presented in this study are included in the article. Further inquiries can be directed to the corresponding author.
